# Prion Protein Paralog Doppel Protein Interacts with Alpha-2-Macroglobulin: A Plausible Mechanism for Doppel-Mediated Neurodegeneration

**DOI:** 10.1371/journal.pone.0005968

**Published:** 2009-06-18

**Authors:** Stefano Benvegnù, Diego Franciotta, Josh Sussman, Angela Bachi, Elisabetta Zardini, Paola Torreri, Cedric Govaerts, Salvatore Pizzo, Giuseppe Legname

**Affiliations:** 1 Scuola Internazionale Superiore di Studi Avanzati - International School for Advanced Studies (SISSA-ISAS), Trieste, Italy; 2 IRCCS, Foundation, Neurological Institute C. Mondino, University of Pavia, Pavia, Italy; 3 Institute for Neurodegenerative Diseases; 4 Department of Neurology, University of California San Francisco, San Francisco, California, United States of America; 5 DIBIT, San Raffaele Scientific Institute, Milan, Italy; 6 Université Libre de Bruxelles, Bruxelles, Belgium; 7 National Centre for Rare Diseases, Istituto Superiore di Sanità, Roma, Italy; 8 Department of Pathology, Duke University Medical Center, Durham, North Carolina, United States of America; University of Oulu, Finland

## Abstract

Doppel protein (Dpl) is a paralog of the cellular form of the prion protein (PrP^C^), together sharing common structural and biochemical properties. Unlike PrP^C^, which is abundantly expressed throughout the central nervous system (CNS), Dpl protein expression is not detectable in the CNS. Interestingly, its ectopic expression in the brain elicits neurodegeneration in transgenic mice. Here, by combining native isoelectric focusing plus non-denaturing polyacrylamide gel electrophoresis and mass spectrometry analysis, we identified two Dpl binding partners: rat alpha-1-inhibitor-3 (α_1_I_3_) and, by sequence homology, alpha-2-macroglobulin (α_2_M), two known plasma metalloproteinase inhibitors. Biochemical investigations excluded the direct interaction of PrP^C^ with either α_1_I_3_ or α_2_M. Nevertheless, enzyme-linked immunosorbent assays and surface plasmon resonance experiments revealed a high affinity binding occurring between PrP^C^ and Dpl. In light of these findings, we suggest a mechanism for Dpl-induced neurodegeneration in mice expressing Dpl ectopically in the brain, linked to a withdrawal of natural inhibitors of metalloproteinase such as α_2_M. Interestingly, α_2_M has been proven to be a susceptibility factor in Alzheimer's disease, and as our findings imply, it may also play a relevant role in other neurodegenerative disorders, including prion diseases.

## Introduction

Prion diseases, generally known as transmissible spongiform encephalopathies or TSE, are fatal neurodegenerative disorders due to the conversion of the cellular form of the prion protein (PrP^C^) into an abnormal, pathogenic and proteinase-resistant form of the same protein (PrP^Sc^). The family of prion diseases comprises Creutzfeldt-Jakob disease (acronym CJD), fatal familial insomnia (acronym FFI), and kuru in humans, chronic wasting disease (acronym CWD), bovine spongiform encephalopathy (acronym BSE), and scrapie in deer, cows and sheep, respectively. Once PrP^C^ is converted into its pathogenic isoform, PrP^Sc^, it accumulates in the brain, and its presence and accumulation is linked to neurodegeneration in affected patients and animals [1], [2].

**Figure 1:**
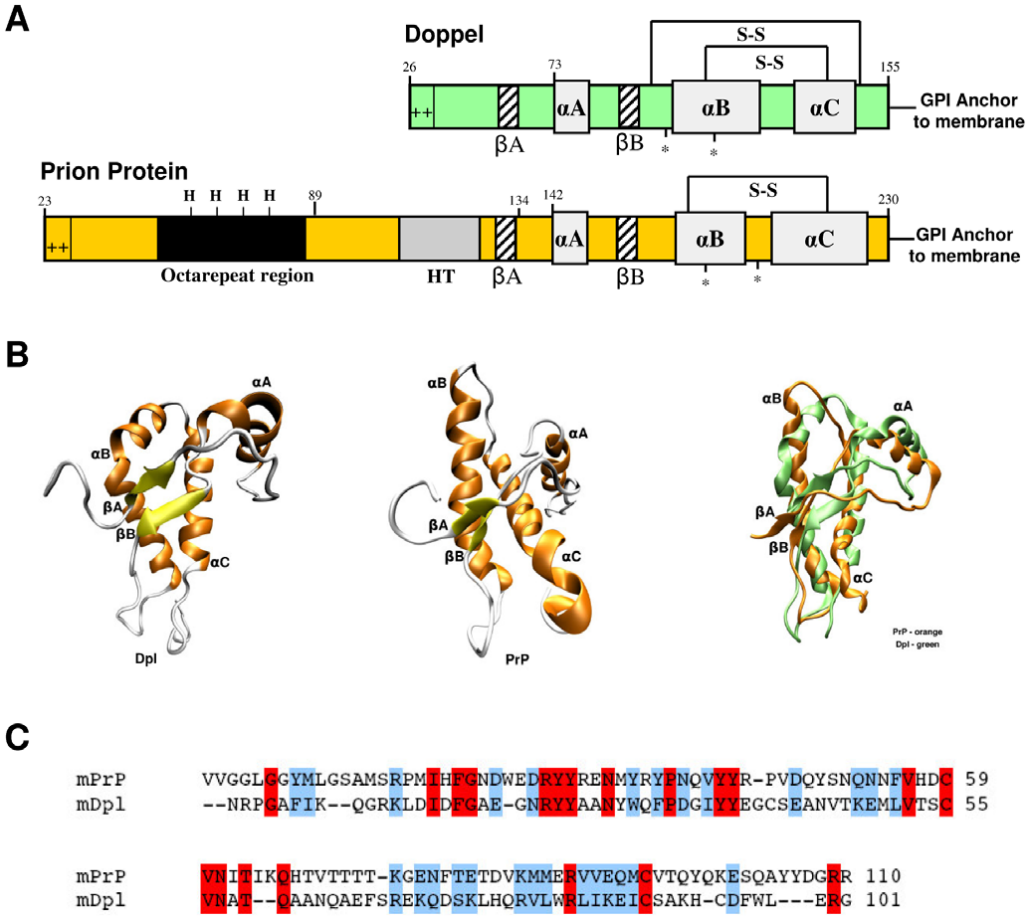
Mature PrP and Dpl protein share common structural architectures. (A) PrP^C^ and Dpl have common secondary structure elements, composed by three alpha helices (αA, αB and αC) and two beta strands (βA and βB). Both PrP^C^ and Dpl have N-glycosylation sites (*), disulfide bridges (S-S) and a GPI-moiety, which links the proteins to the extracellular side of the cellular membrane. PrP^C^ and Dpl also share a positively charged N-terminus. PrP^C^ contains five octapeptide repeats capable of binding copper through histidine residues (modified from [52]). (B) The topology of Dpl (PDB code: 1I17, left structure) is very similar to that of PrP^C^ (PDB code: 1AG2, central structure). A significant difference is that αB helix of Dpl (in green, right image) is bent and that the two beta strands are oriented differently than those in the PrP^C^ (in orange, right image). (C) Sequence alignment between mouse PrP^C^ (mPrP, residues Val^120^–Arg^229^; SwissProt entry: P04925) and mouse Dpl (mDpl, residues Asn^55^–Gly^155^; SwissProt entry: Q9QUG3) proteins. In this tract the two proteins share 18% of sequence identity and 44% of sequence similarity. Fully conserved residues are highlighted in red, while semi-conserved are shown in blue.

In recent years, doppel protein (Dpl), a PrP^C^ paralog, has been identified as a protein sharing common biochemical and structural properties with the latter [3], [4], [5]. Dpl and the C-terminal domain of PrP^C^ have only approximately 25% of primary aminoacidic sequence identity (Figure 1C), yet their tertiary structure is very similar (Figure 1B), and both share the same secondary structure elements consisting of a three α-helix bundle with two short β-strands (Figure 1A) [5]. Like PrP^C^, Dpl has two N-glycosylation sites, and a highly enriched basic aminoacids flexible amino-terminal region which likely contributes to its cellular trafficking (Figure 1A). However, in contrast to PrP^C^, Dpl is expressed at very low levels in the mouse central nervous system (CNS), whereas its expression is high in non-nervous tissues, e.g. testes. Notably, two transgenic (tg) mouse (Mo) lines ablated for the PrP gene develop late-onset ataxia as well as Purkinje cells and granule cells degeneration in the cerebellum [6], [7]. In these tg lines, Dpl is ectopically upregulated in the CNS. In contrast, other PrP-knockout murine lines, in which Dpl ectopic expression in the CNS is absent, do not develop either ataxia or neurodegeneration. Furthermore, Dpl levels in the CNS proved to be inversely correlated to the onset age of ataxic phenotype [8]. Interestingly, tg mice expressing PrP with amino-proximal deletions (named PrPΔF) show ataxia and degeneration of the cerebellar granule cell layer within a few weeks after birth [9]. PrPΔF mutants lack regions absent also in Dpl, therefore sharing structural properties with the latter. Restoration of wild type PrP presence in the CNS of mice expressing either Dpl [8] or PrPΔF [9] rescues the ataxic phenotype. These findings suggest that Dpl expression may lead to neurodegeneration similar to truncated PrP, and that the wild type PrP^C^ and Dpl may have opposite and antagonistic functions. In fact, cell surface PrP^C^ may have a protective role and antagonize the toxic effect of Dpl in the CNS, either by interacting directly with Dpl, or *via* another protein, or *via* non competitive mechanisms [10]. Indeed, a neuroprotective function for PrP^C^ has been proposed [11], [12], [13].

In order to investigate the possibility that PrP^C^ and Dpl may have common binding partner(s), we previously described novel constructs of PrP^C^ and Dpl fused to the Fc region of human IgG1, and used these fusion proteins as probes to stain sections of mouse brain [14]. We found restricted binding of both these fusion proteins to the granule cell layer (GCL) of the cerebellum, indicating the presence of ligands in this region that specifically bind to either PrP^C^ or Dpl. These findings prompt us to explore several biochemical routes to identify physiological interacting molecules of PrP^C^ and Dpl in the cerebellum. However, while many physiological and putative PrP interacting partners have been described (reviewed in [15]), very little is known about Dpl-interacting proteins. We therefore focused on the identification of Dpl binding proteins.

We utilized native isoelectric focusing (IEF) plus non-denaturing polyacrylamide gel electrophoresis of rat cerebellum slices to extract and isolate the protein ligands of Dpl. These techniques proved to be essential in our quest because they ensured that the isolated proteins retained their native folding. By mass spectrometry (MS) analysis, we demonstrated unequivocally that rat alpha-1-inhibitor-3 (α_1_I_3_) is a major ligand for Dpl in the brain. Rat α_1_I_3_ is a member of the alpha-macroglobulin superfamily, which contains both proteinases inhibitors and complement molecules [16]. By homology search, we also identified mouse and human alpha-2-macroglobulin (α_2_M) as Dpl interacting molecules. Interestingly, PrP^C^ was not found to interact to any alpha-2-macroglobulins, suggesting that this class of proteins may not be shared ligands for both PrP^C^ and Dpl. Finally, in this study we also demonstrate a strong interaction between PrP and Dpl, indicating an intriguing plausible molecular mechanism for their biological antagonism.

## Materials and Methods

### Animals

Animal husbandry was performed in compliance with the European laws [European Community Council Directive, November 24, 1986 (86/609/EEC)], and in accordance to the guidelines of the San Raffaele Hospital Institutional Animal Care and Use Committee.

### Proteins

Apo-transferrin, mouse albumin, aprotinin and alpha-chymotrypsin were purchased from Sigma-Aldrich (St. Louis, MO, USA).

### Recombinant mouse PrP and Mouse Dpl production and purification

The production and purification of recombinant proteins are described in Text S1.

### Cloning and production of MoPrP-Fc and MoDpl-Fc fusion proteins

MoPrP-Fc and MoDpl-Fc were produced as previously described [14].

### Extraction and identification of the protein from cerebellar tissue with non-denaturing separation techniques

#### Analytical IEF

**Figure 2:**
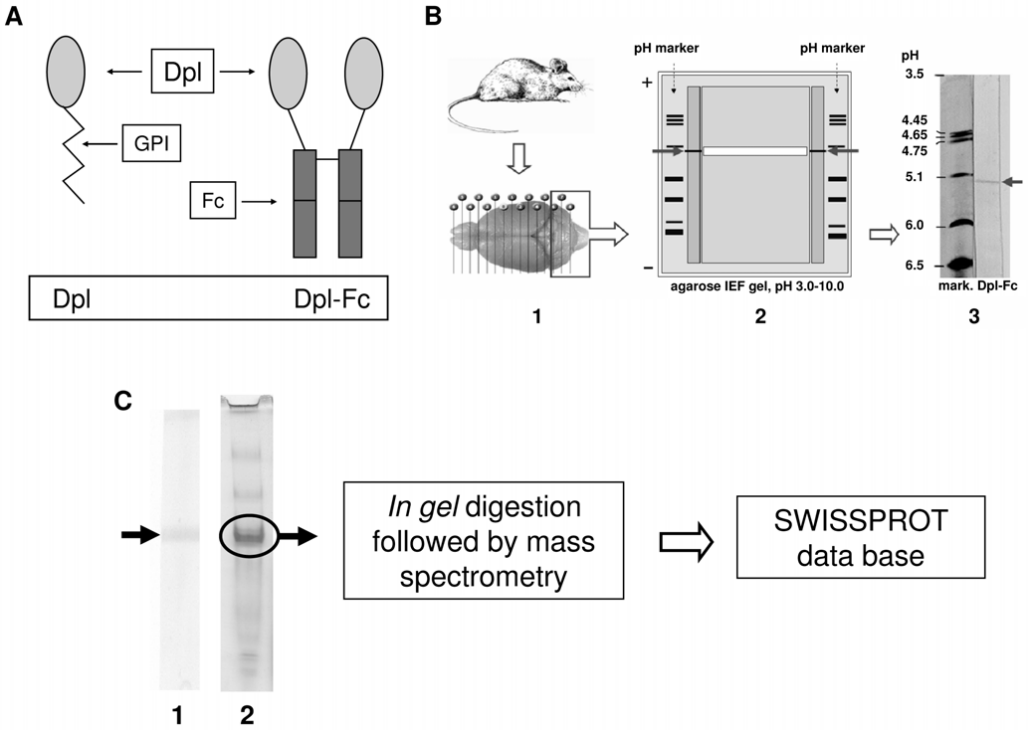
Identification of Dpl interacting protein in rat cerebellar slices. (A) Schematic representation of Dpl-Fc construct. Dpl protein is fused with the Fc region of a human immunologlubulin IgG1. The resulting fusion protein is denominated Dpl-Fc. (B) Direct tissue isoelectric focusing of solid tissues technique. This method allows the extraction of more numerous protein *vs* extracts of tissue homogenates [17]. The technique also prevents the loss of conformational epitopes, which are denatured by standard extraction procedures. Cryostatic rat cerebellar slices (B-1) were put onto the gel. After IEF run, two lateral parts of the gel were blotted onto nitrocellulose stripes, and tested with MoDpl-Fc, for the identification of the binding protein (B-2). Arrows indicate the band (B-2 and B-3). Identification of the band's approximate pI, deduced by comparison with the pH marker, was 5.3. The central part of the gel was used for the protein recovery: a strip of the agarose gel (in white, B-2), which comprised the band (arrows), was excised and placed inside a microtube in PBS. After incubation and centrifugation, the sample was run on native gradient PAGE (C). (C) Gel electrophoresis of recovered protein. To yield amounts of the protein of interest suitable for proteomics, the sample obtained by isoelectric focusing analysis was split into two aliquots and run on PAGE. One aliquot was transferred onto nitrocellulose paper and tested with MoDpl-Fc to identify the protein of interest (C-1), the other one was silver stained (C-2). The arrow indicates the protein that binds to MoDpl-Fc, which was eventually recognized to be rat α_1_I_3_ precursor.

The technique for direct tissue isoelectric focusing (IEF) has been previously described elsewhere [17]. For brain tissue IEF, cerebella from Sprague-Dawley rats were taken, immediately frozen in an isopentane-filled tube cooled in liquid nitrogen, and stored at −80°C until use. Sections of 30 µm thickness were cut on a cryostat microtome and, still frozen, applied onto the hydrophilic side of a 1.0×10.0 cm plastic support (GelBond, FMC, Rockland, ME, USA) (Figure 2B-1 and Figure 2B-2). The support was immediately placed upside down onto an agarose gel plate for IEF (pH range, 3.0–10.0, Cambrex, Rockland, ME, USA; IEF apparatus: Resolve Omega, Isolab Inc., Akron, OH, USA) and located both centrally and near the cathod. A pH marker (IEF Standards, Bio-Rad, Hercules, CA, USA) was electrophoresed at the two lateral sides of the plastic support. Focusing was at 12 W, constant power, to a maximum of 700 V for 2 h at 9°C. The plastic support and tissue sections were removed after 30 min. At the end of the run, the gel was cut to obtain a large central part, which was used for the preparative procedure, and two small lateral parts, which included the two lanes with pH marker, and the adjacent, 0.8 cm wide lanes where the proteins had run. The lanes with pH marker were silver stained [18]. The adjacent lanes were blotted onto nitrocellulose [19]. Briefly, a sheet of nitrocellulose paper (Schleicher & Schuller, Keene, NH, USA) was laid onto the gel, and covered with a PBS-wetted sheet of fine filter paper, 8 sheets of absorbent paper, a glass plate, and a weight of 0.5 kg for 1 h. Each of the following steps was performed at 4°C, with gentle agitation, and alternated by washing with PBS. The nitrocellulose was saturated in 10% bovine serum albumin in PBS for 1 h. Incubated first with non-diluted medium containing MoDpl-Fc (6 mL) overnight, then with a biotinylated anti-human Fc antibody (Dako, Glostrup, Denmark) at a dilution of 1∶500 in 2% bovine serum albumin in PBS for 1 h. The treated nitrocellulose was developed with avidin-biotin (Vectastain ABC kit, Vector, Burlingame, CA, USA) and stained with 0.5 mg/mL of diaminobenzidine in PBS and 0.02% of H_2_O_2_ up to visualize the band. The reaction was stopped with PBS.

#### Preparative IEF

The central part of the gel was used for protein recovery (Figure 2B-2). Analytical IEF indicated the position where the protein migrated in the gel (isoelectric point, pI). To be sure of including the desired protein, an horizontal piece of the gel, spanning 2 mm above and below protein's migration point, was removed from the support, and put into a conical minitube in PBS (200 µL). After an overnight shaking at 4°C, the minitube was centrifuged at 7,000×g for 1 min. The liquid, which contained the protein recognised by MoDpl-Fc, was recovered (∼100 µL), and concentrated (2×) with a vacuum desiccator. In the sample, the searched protein was mixed with other proteins with similar, or slightly different pI. To obtain one single-band protein, which was concentrated and suitable for mass spectrometric (MS) identification, the sample was split into two aliquots of 25 µL and run in electrophoresis on a non-denaturing polyacrylamide gradient gel (4–15% acrylamide; Mini-Protean II; Bio-Rad, Hercules, CA, USA) at 100 V for 3 h. One lane of the gel was electrically blotted onto nitrocellulose paper (Mini Trans-blot chamber, Bio-Rad, Hercules, CA, USA) in Tris/glycine buffer at 200 mA for 3 h at 4°C. The nitrocellulose was then treated as previously described after analytical IEF to confirm binding specificity, and estimate the point of the protein migration. This point was used to identify the protein in the second lane, which was silver stained with a protocol suitable for MS sequencing [20] (Figure 2C).

### Protein identification by MALDI-TOF MS analysis

Gel bands were manually excised, reduced, alkylated and digested overnight with bovine trypsin (Roche, Basel, Switzerland) as described elsewhere [20]. One µL aliquots of the supernatant were used for MS analysis on a MALDI-TOF Voyager-DE STR (Applied Biosystems) mass spectrometer. Spectra were accumulated over a mass range of 750–4,000 Dalton (Da) with a mean resolution of about 15,000. Spectra were internally calibrated using trypsin autolysis products and processed *via* Data Explorer software version 4.0.0.0 (Applied Biosystems). Alkylation of cysteine by carbamidomethylation, and oxidation of methionine were considered as fixed and variable modifications respectively. Two missed cleavages per peptide were allowed, and a mass tolerance of 50 ppm was used. Peptides with masses correspondent to those of trypsin and matrix were excluded from the peak list. Proteins were identified by searching against a comprehensive non redundant protein database (NCBInr 20090222; Taxonomy: Rodentia) using MASCOT algorithm [21].

### Homology search

Initial sequence similarity searches were performed with BLAST [22] using the α_1_I_3_ sequence as probe. All significant hits returned are part of the proteinase-binding α-macroglobulins family. Sequence alignments were performed using ClustalW [23] on a non-redundant set of the mammalian sequences from this protein family. A similarity tree was generated using TreeView [24].

### Preparation of α_1_I_3_ and α_2_M

The protein α_2_M was purified to apparent homogeneity by Zn^2+^-chelate chromatography, as previously described [25], with minor modifications [26].

Rat α_1_I_3_ was purified to apparent homogeneity, as previously described [26].

### Enzyme-Linked ImmunoSorbent Assay (ELISA)

A general ELISA protocol was used with α_1_I_3_, α_2_M, MoDpl, MoPrP, MoPrP-Fc, apo-transferrin, mouse albumin, aprotinin and alpha-chymotrypsin. In detail, 96 well ELISA plates (Dynex Corporation, Chantilly, VA, USA) coated with the various proteins were used in biochemical assays for verification of protein:protein interaction. A conventional ELISA procedure was used with a slight modification [27]. For each well, stated amount of protein was diluted in 100 µL of 0.1 M sodium bicarbonate solution, pH 8.6 and incubated overnight at 4°C. After nine washes with 1×TBST, the well was blocked using saturating solution [0.25% BSA, 0.05% Tween-20 in Dulbecco's PBS Ca^++^ Mg^++^ Free (Gibco, Invitrogen, Carlsbad, CA, USA)] for 1 h at room temperature (RT). The addition of proteins to the wells was preceded by a pre-incubation in saturating solution at 4° C for 1 h. All incubations for protein binding were performed at RT after this step. In general, indicated amounts of PrP were diluted in saturating solution and incubated for 2 h. Nine repeated washes between incubations were performed with 1×TBST. For PrP detection, either 2 µg/mL of humanized anti-MoPrP HuM-D18 antibody fragments Fab [28] or a 1∶500 dilution of a rabbit polyclonal anti-MoPrP R073 [29] was added and incubated for 1 h at RT. For Dpl detection, a 1∶1,000 dilution of a rabbit polyclonal anti-MoDpl was added and incubated for 1 h at RT [8]. A 1 h incubation with 100 µL of goat anti-human Fab (1∶1,000 dilution) conjugated to alkaline phosphatase (Pierce, Rockford, IL, USA) or 100 µL of an anti-rabbit IgG-Alkaline Phosphatase 1∶5,000 (Promega Corporation, Madison, WI, USA) were added for 1 h at RT. MoPrP-Fc was detected using a goat polyclonal anti-human Fc antibody at 1∶1,000 ratio and incubated for 1 h at RT.

### Surface Plasmon Resonance

Surface Plasmon Resonance (SPR) analysis was performed at 20°C using the Biacore 2000 biosensor system (Biacore AB, Uppsala, Sweden). For surface preparation, recDpl was immobilized on a carboxymethyldextran (CM5; Biacore AB) sensor chip using standard amine-coupling chemistry. EDC (N-ethyl-N'-[3−(dimethylamino)−propyl] carbodiimide) and NHS (N-hydroxysuccinimide) were used for surface activation and ligand coupling; ethanolamine was used for deactivation. For kinetic analysis, duplicate injections of analytes [recMoPrP(89–230) and recMoPrP(23–230)] in various concentrations (0.44–4.4 µM) were run under the buffer condition of HBS-N (10 mM HEPES, 150 mM NaCl), pH 7.4 (Biacore AB). Analytes were injected over the Dpl-coupled and uncoupled surfaces in a CM5 sensor chip at a flow rate of 20 µL/min for 2 min. Dissociation was monitored for 5 min. SPR from the uncoupled surface was used as a reference. As many as four independent serial dilutions were carried out for each binding experiment. The obtained kinetic data were analyzed by BIAevaluation software (Biacore AB).

### Statistical analyses

Results are given as mean±SD. Statistical analyses were performed by using Student's *t* test (two-tailed), and the null hypothesis was rejected at the 0.05 level.

## Results

### MoPrP(23–230) and MoDpl(26–155) expression and purification in *E.coli*

The production of pure and natively folded of either recMoPrP(23–230) or recMoDpl(26–155) has been described in [30] and [8], respectively.

### MoPrP-Fc and MoDpl-Fc expression in the N2a cell line

MoPrP(23–231) and MoDpl(26–155) sequences were inserted into an expression plasmid containing the Fc region of human IgG1 to produce the MoPrP-Fc and MoDpl-Fc constructs, respectively.

The resulting plasmids were transfected independently in the mouse neuroblastoma N2a cell line and the proteins were expressed and secreted in the supernatant. Both MoPrP-Fc and MoDpl-Fc were expressed at comparable levels as dimers as detected by Western blot (data not shown), and used at concentration of 20 µg/mL. The dimerization is conferred by the Fc portion and neither by MoPrP nor MoDpl. A control Fc protein was also expressed and successfully secreted in the supernatant of N2a cells (data not shown). Further characterization of these constructs has been described in detail elsewhere [14]. A schematic representation of Dpl-Fc construct is shown in Figure 2A.

### IEF of brain proteins and identification of α_1_I_3_ as Dpl-interacting partner

**Figure 3:**
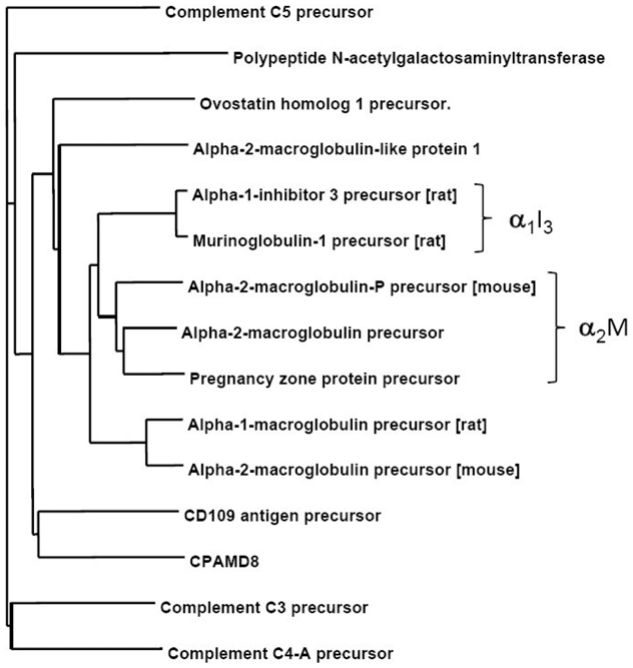
Similarity tree of a non-redundant set of the mammalian members of the α_2_M type of proteinase inhibitors. Except where noted, the human sequences were taken for alignment purposes. The α_2_M and rat α_1_I_3_ families are respectively highlighted by right braces.

After identifying the presence of MoDpl-Fc binders in the GCL of the cerebellum of mice [14], we attempted to purify the specific protein, or proteins, responsible for such positive reactivity. To this purpose we employed rat cerebella. After extraction and separation of native rat cerebellar proteins with IEF, we transferred the protein to nitrocellulose support paper and used MoDpl-Fc as probe. MoDpl-Fc proved to bind a sharp band at pH 5.3 (Figure 2B-3). Subsequent preparative procedures (see Materials and Methods) allowed us to obtain amounts of the detected protein suitable for mass spectrometry analysis, which identified the band as rat α_1_I_3_ (Figure 2C and Figure S1) (gi/83816939, 29 matched peptides out of 39, sequence coverage 26%, Score: 231 Expect: 1.8e-018).

### Identification of α_2_M by homology search

As α_1_I_3_ is a monomeric protein member of the alpha-macroglobulin superfamily [31], we investigated the possibility that Dpl could also interact with other members of the macroglobulins family whose expression is not restricted to rat. To assess this hypothesis, we performed a sequence homology search analysis and identified also mouse and human α_2_M as potential interacting partners of Dpl. Figure 3 shows a similarity tree representing a multiple sequence alignment of a non-redundant set of the mammalian sequences from the proteinase-binding α-macroglobulins family of proteins, which are large glycoproteins found on the plasma of vertebrates that can inhibit proteinases from all catalytic classes by a molecular trapping mechanism [32]. They contain a conserved thiolester motif that allows rapid classification. In addition to the proteinase inhibitors, the family also contains the complement components C3, C4 and C5.

As shown in Figure 3, the members of the family showing the strongest similarity to α_1_I_3_ are the α_2_M proteins, which prompted experimental testing of such proteins as potential Dpl interacting partners (see below).

### ELISA validation of Dpl binding to α_1_I_3_

**Figure 4:**
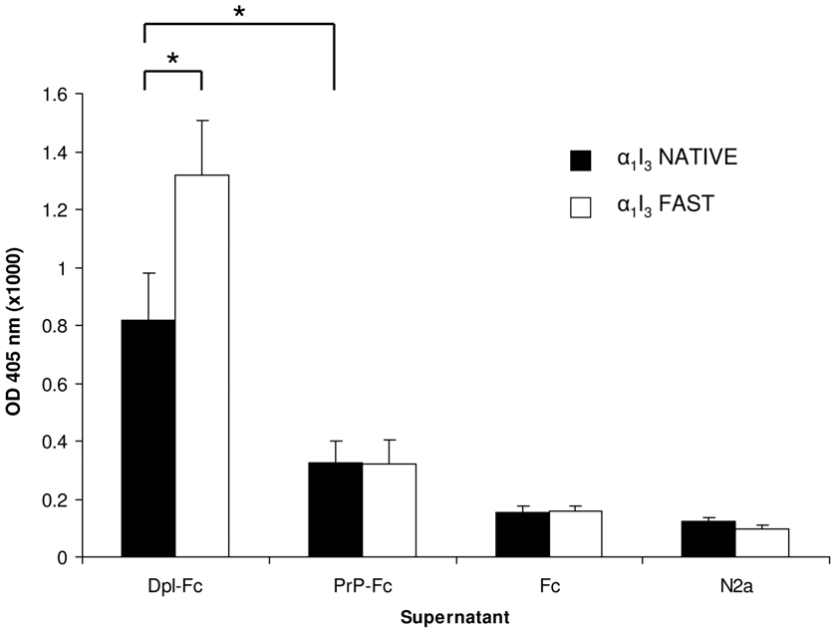
Dpl binds to α_1_I_3_. ELISA plates coated with α_1_I_3_ in its native (black bars) or activated, fast (white bars) forms were incubated with the supernatant of respectively MoDpl-Fc transfected N2a cells (Dpl-Fc), MoPrP-Fc transfected N2a cells (PrP-Fc), mock-transfected N2a cells (Fc) and non-transfected N2a cells (N2a). *, *p*<0.05. Data shown are representative of at least three independent experiments.

Dpl binding to α_1_I_3_ was investigated by ELISA. The assay was performed using microtiter plates coated either with native (NATIVE) or with methylamine-activated (FAST) form of α_1_I_3_, and then incubated with the supernatant of respectively un-trasfencted, mock-transfeted, and either MoPrP-Fc or MoDpl-Fc transfected N2a cells. The results of this assay show a significant binding signal for α_1_I_3_ by the conditioned medium of Dpl-Fc transfected N2a cells (Figure 4).

Indeed, the ‘FAST’, activated form of α_1_I_3_ achieved a greater binding signal to Dpl-Fc if compared to the signal obtained using ‘NATIVE’ α_1_I_3_.

On the contrary, a much weaker signal was detected for α_1_I_3_ incubated with the supernatant of PrP-Fc transfected cells, being the former either in the native or in the activated form. Thus, binding of α_1_I_3_ to PrP seems unlikely.

### Confirmatory ELISA for α_2_M

After the identification of rat α_1_I_3_ by IEF and MS (see previous sections), *via* bioinformatic analysis we also identified mouse and human α_2_M as a potential Dpl-interacting partner. In order to experimentally validate this hypothesis, α_1_I_3_ and α_2_M were coated onto microtiter plates and subjected to ELISA, both in their ‘NATIVE’ form and in their activated form, and then incubated with recDpl (see Materials and Methods).

**Figure 5:**
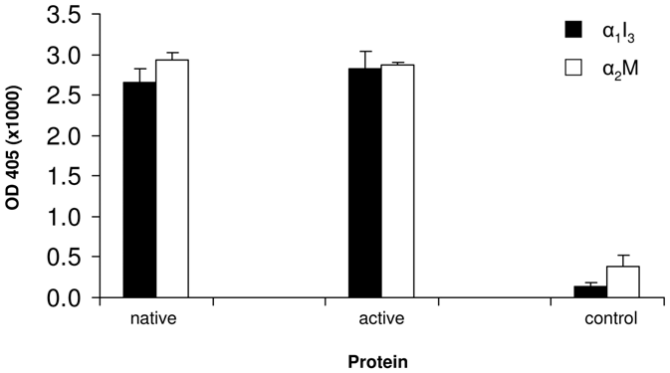
ELISA binding measurements of recDpl with α_2_M and α_1_I_3_. Equimolar amounts of α_2_M and α_1_I_3_ were coated onto ELISA plates both in their native and in their active form, and then incubated with recDpl. Primary antibody was used as control: black bars, α_1_I_3_; white bars, α_2_M. Data shown are representative of at least three independent experiments.

As expected, the binding signal of α_2_M to recDpl was comparable and generally equivalent to the one of α_1_I_3_. No statistically different binding signals were achieved both by the native and the activated forms of α_1_I_3_ and α_2_M (Figure 5). This result seems contradictory with the results previously discussed, in which native and activated forms show different binding capability, but this variation may be due to the differences between the monomeric, unglycosylated, prokaryotic-produced recDpl and the dimeric, glycosylated, eukaryotic-produced Dpl-Fc.

In addition, several proteins such as apo-transferrin, mouse albumin, aprotinin and alpha-chymotrypsin were tested in ELISA in order to validate the specificity of recDpl interaction with α_2_M. In three independent experiments, no detectable recDpl binding to either apo-transferrin, or mouse albumin, or aprotinin or alpha-chymotrypsin was found (data not shown).

### Saturation of binding of α_2_M by Dpl

**Figure 6:**
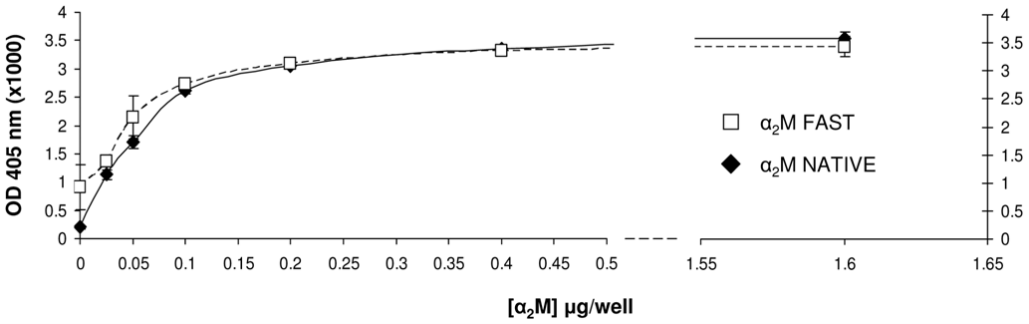
Saturation curve for recDpl with increasing α_2_M concentrations. The working range of coated-α_2_M was from 25 ng to 1.6 µg/well. recDpl was added 1.0 µg/well. A trend of linearity is observed for values up to 100 ng/well, whereas a trend of plateau can be found for values higher than 400 ng/well. No difference is observed in the binding to recDpl comparing the native form with the activated form of α_2_M. *y* axis, OD values at 405 nm (x1,000); *x* axis, µg of proteins per well; white squares, α_2_M “fast” form; black rhombi, α_2_M “native” form.

In order to verify that the binding of α_2_M to Dpl was saturable, we carried out a titration by means of ELISA. As shown in Figure 6, the binding of Dpl (1.0 µg/well) can be saturated applying increased concentrations of both native and activated α_2_M (working range from 25 ng/well to 1.6 µg/well).

### Measuring Dpl and PrP interaction

**Figure 7:**
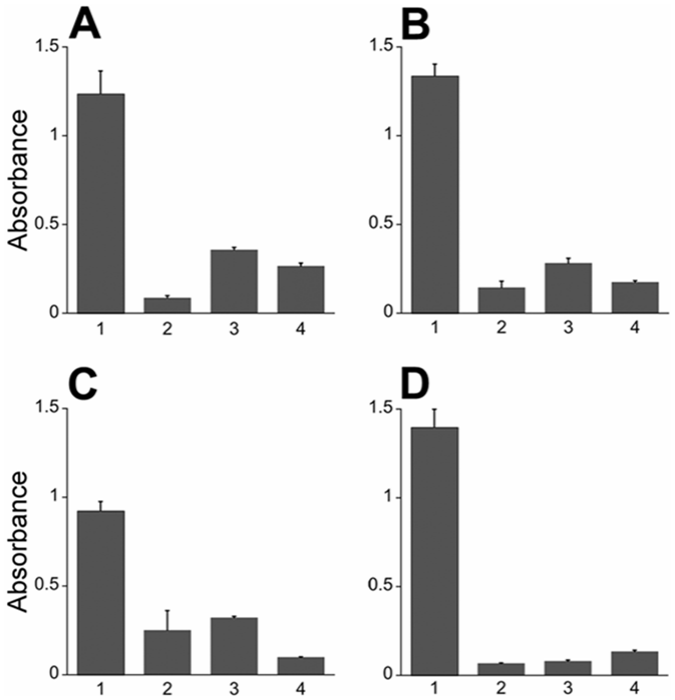
recDpl and recPrP bind to each other. To determine protein interaction, the primary protein was coated onto a 96-well plate then incubated with the secondary protein. After washing, the presence of the secondary protein was detected by ELISA (Column 1). Control experiments were performed without the primary protein (Column 2), secondary protein (Column 3), or primary antibody (Column 4). (A) Full-length recPrP(23–230) coated onto the plate with recDpl as the secondary protein. Dpl binding was detected using a rabbit polyclonal antibody to Dpl. (B–D) recDpl was coated onto ELISA plates and either recPrP(23–230) (B), recPrP(89–230) (C) or PrP-Fc (D) were incubated and measured for binding. PrP molecules were detected using either a rabbit polyclonal antibody to PrP (B, C) or with an anti-human Fc secondary antibody (D). All ELISA measurements were completed using an AP detection system at 405 nm. *y* axis, OD values at 405 nm (×1,000). For all panels, graphs represent mean (bar) and standard deviation (error bar) from measurements of at least three independent experiments.

Although the molecular events underlying PrP-mediated rescue of Dpl neurotoxicity are not well understood, one model suggested for this interaction is the binding of PrP to Dpl. We performed ELISA experiments to determine whether recPrP and recDpl bind to each other. Indeed, recDpl bound to immobilized recPrP(23–230) in a direct ELISA, detected using a rabbit polyclonal antibody to Dpl (Figure 7A). Both recPrP(23–230) and recPrP(89–230) bound to immobilized recDpl, as detected by a rabbit polyclonal antibody to PrP (Figure 7B and Figure 7C). Full-length recPrP(23–230) showed a greater binding capacity to Dpl compared to recPrP(89–230), which lacks the octapeptide repeat region. Moreover, a dimeric form of PrP expressed in N2a cells, PrP-Fc [14], also bound to Dpl as effectively as monomeric, full-length recPrP(23–230) (Figure 7D).

**Figure 8:**
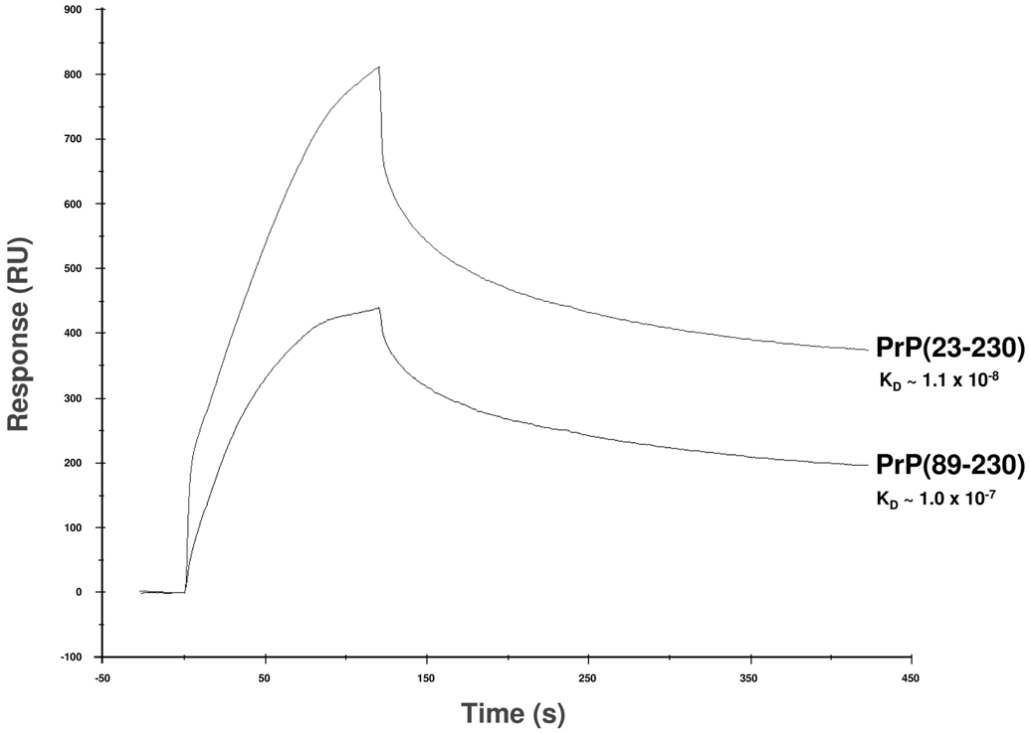
SPR sensorgram comparison of the kinetics of binding between Dpl with either recombinant full-length PrP (PrP(23–230)) or truncated PrP (PrP(89–230)). Equimolar amounts of PrP(23–230) and PrP(89–230) were injected over Dpl-coupled sensor chip, and kinetics of binding was monitored as response units (RU, *y* axis) on time (seconds, *x* axis). Full-length PrP(23–230) proved to possess higher binding capability for Dpl than truncated PrP(89–230). The sensorgrams in the figure were obtained with injection of 800 nM of both PrP(23–230) and PrP(89–230).

To understand the binding between Dpl and PrP molecules further, we analyzed the kinetics of binding between these proteins using Surface Plasmon Resonance (SPR) biosensor technology. The binding data were analyzed by a mathematical calculation based on the 1∶1 (Langmuir) binding model (Table 1). This simulation perfectly matched with our experimental data, which demonstrated a 1∶1 interaction between Dpl and both PrP(23–230) and PrP(89–230). Similar to results revealed by the previously performed ELISA, the binding capability of Dpl was greater with full-length recPrP(23–230) than with recPrP(89–230) (Figure 8). The K_D_ of Dpl-PrP binding was ∼10 times smaller with full-length PrP than with MoPrP(89–230) (Table 1). As suggested by k*_on_* and k*_off_* constants of each binding, this was mainly due to the faster association rate of recPrP(23–230) to Dpl compared to that of recPrP(89–230); the dissociation rates did not differ greatly (Figure 8 and Table 1).

**Table 1:** Kinetics analysis of interaction between PrP and Dpl using SPR.

	recPrP(89–230)	recPrP(23–230)
**k** ***_on_*** ** (M^−1^s^−1^)**	(8.4±0.02)×10^3^	(1.2±0.08)×10^5^
**k** ***_off_*** ** (s^−1^)**	(8.7±0.12)×10^−4^	(1.3±0.034)×10^−3^
**K_D_ (M)**	**1.0×10^−7^**	**1.1×10^−8^**

## Discussion

A major physiological function for PrP^C^ is still elusive and it is becoming increasingly clear that PrP^C^ may play a pleiotropic role in a variety of cellular functions. Studies have been conducted on several strains of mice devoid of PrP^C^, but these animal models have not been able to clarify PrP^C^ actual functions. In fact, in certain PrP-knockout mouse strains the PrP^C^ paralog Dpl was identified and emerged as a protein whose brain expression may alter CNS functions. Indeed, neurodegeneration of Purkinje and granule cells of the cerebellum occurs when Dpl is ectopically expressed in the brain [3]. The level of expression of Dpl in the brain is inversely correlated to the age at which Purkinje and granule cells degeneration, as well as ataxic signs, start to be observed. The concomitant expression of full-length PrP and Dpl in tg mice is able to counteract the Dpl-induced ataxic phenotype, suggesting that the expression of PrP can neutralize the toxic effect of Dpl either by interacting directly with Dpl or through another protein [8]. Hence, we endeavored to search for Dpl interacting protein(s) in the cerebellum, in order to better understand the role of Dpl in cerebellar neurodegeneration. By using Dpl-Fc as a tool to probe brain histoblots, we firstly identified a restricted expression of Dpl binding partners in the granule cells of either wild-type or PrP^C^-devoid mice [14]. Here, by combining IEF of rat cerebellar proteins and mass spectrometry analysis, we demonstrated for the first time the identification of rat α_1_I_3_ as Dpl interacting partner in the cerebellum. Rat α_1_I_3_ is a plasma proteinase inhibitor, which belongs to the superfamily of the alpha-macroglobulin proteins (Text S2). We then performed sequence homology analysis searches in order to find proteins having high sequence homology with α_1_I_3_ not restrictedly expressed in rat. We identified human α_2_M, a member of the inhibitory macroglobulin family, and further confirmed the binding of α_2_M to Dpl as well.

The inhibitory capacity of the alpha-macroglobulins resides in their ability to entrap proteinase molecules and thereby hinder the access of high molecular weight substrates to the proteinase active site. This ability is thought to require at least two alpha-macroglobulin subunits, yet the monomeric alpha-macroglobulin rat α_1_I_3_ also inhibits proteinases [16]. Macroglobulins circulate in blood as an inactive precursor, which is activated by a proteinase after proteolytic attack on the so-called “bait region”. Subsequently, thiol ester bonds present in each α_2_M subunit are activated and generally the proteinase incorporates into the Glx residue exposed by these events. The covalent binding by the macroglobulin of the proteinase causes a conformational change in the former which is responsible for the electrophoretic shift of the activated form, thus referred to as “fast”, if compared to the inactive, “slow” form, or “native” form, of the macroglobulin. The activation of the macroglobulin can also be achieved *in vitro* by methylamine treatment. Activated forms of α_2_M are rapidly removed from the circulation by cellular receptors, main example of this is the low-density lipoprotein receptor-related protein (LRP), which recognizes the “fast” but not “slow” form of α_2_M [16].

recDpl proved to bind α_2_M with generally the same apparent affinity as for α_1_I_3_. To explore whether the binding of Dpl to α_1_I_3_ and α_2_M could be discriminative between the inactive and the activated form of the proteins, binding assays were performed using the two distinct biological forms. However, apparently discordant data emerged in ELISA tests when using either recDpl as antigen, or when using Dpl-Fc. There was no significant difference in the binding of recDpl to both the inactive and the activated form of α_1_I_3_ and α_2_M, whereas Dpl-Fc showed a significant higher binding activity for the “fast” form of α_1_I_3_ in contrast to the “native” form. However, while recDpl is bacteria-produced in monomeric and unglycosylated form, Dpl-Fc is produced by transfection of eukaryotic N2a cells and is secreted in a dimeric and glycosilated form. Therefore, further investigations are needed to clarify whether there could be any significant difference in binding between Dpl and α_1_I_3_ and α_2_M *in vivo*.

Dpl shares common biochemical and structural features with PrP^C^, i.e. it is a GPI-anchored protein, can bind divalent copper ions, and folds in a PrP^C^-like tertiary structure [4], [5]. Therefore, we tested whether PrP^C^ would also interact with α_1_I_3_ and α_2_M, but this was not the case, as PrP^C^ shows no binding signal when compared to Dpl (Figure 4 and Figure 5).

These results suggest that macroglobulins may not be common interacting partners both for PrP and Dpl. Differently from PrP^C^, Dpl is not physiologically detectable in the adult mouse brain, and the level of Dpl messenger RNA in the CNS is under strictly regulated developmental control [33]. Thus, we suggest that Dpl interaction with α_1_I_3_ and/or α_2_M may not be an usual physiological feature in the brain. However, this interaction might shed light on the role of Dpl on the cerebellar neurodegeneration occurring in tg mice lines, such as *Ngsk* and *Rcm0*, which ectopically overexpress Dpl in their CNS [3], [6].

**Figure 9:**
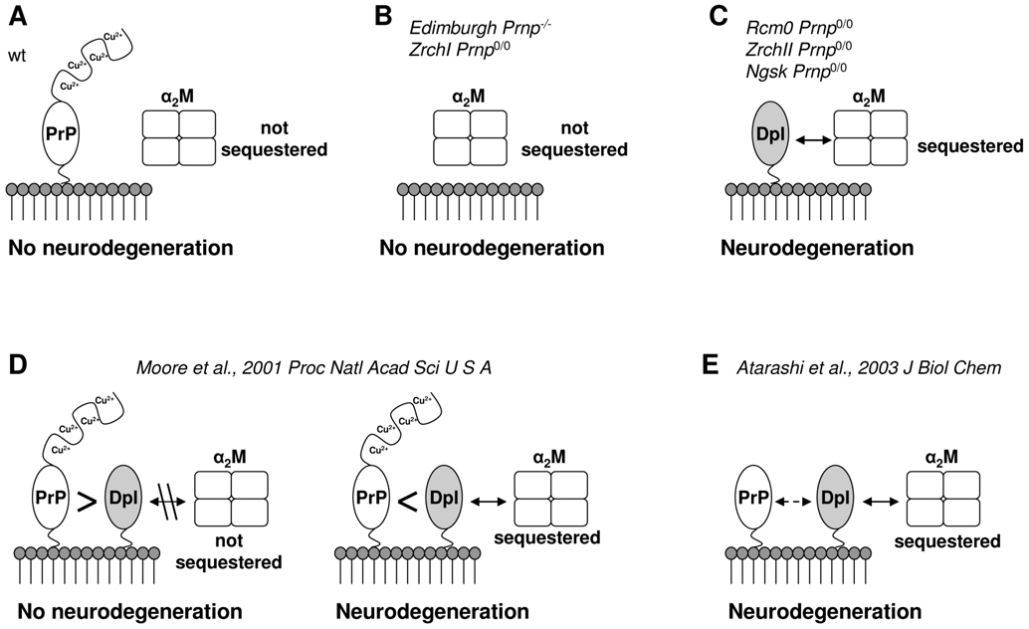
Dpl-mediated model of cerebellar neurodegeneration. The model bears on the postulations as follows: (A) PrP and α_2_M do not interact each other, therefore α_2_M is physiologically regulated in the cerebellum; (B) in *Prnp* ^0/0^ mice, α_2_M is still under physiological regulation as in wild type situation; (C) in absence of PrP and in simultaneous presence of Dpl, α_2_M is sequestered and deregulated, thus leading to cerebellar neurodegeneration. (D) PrP and Dpl are co-expressed, bind and antagonize each other depending on their stoichiometric ratio: on the left, PrP levels are higher than Dpl, PrP sequesters the entire amount of Dpl and thus prevents Dpl interaction with α_2_M; on the right, Dpl expression is higher than PrP, and residual amounts of Dpl unbound to PrP are still capable of binding α_2_M. (E) N-terminally truncated PrP binds with less affinity to Dpl, thus permitting it to bind α_2_M. Mouse models are cited as follows: ZrchI *Prnp* ^0/0^ [53]; Edimburgh *Prnp* ^-/-^ [54]; Rcm0 *Prnp* ^0/0^ [3]; ZrchII *Prnp* ^0/0^ [55]; Ngsk *Prnp* ^0/0^ [6].

In light of our findings, we propose a novel possible mechanism where Dpl-induced cerebellar neurodegeneration may be due to withdrawal of natural inhibitor(s) of metalloproteinases, such as α_1_I_3_ and α_2_M (Figure 9). The absence of neuropathological signs in the cerebellum of both wild type (Figure 9A) and *Prnp* ^0/0^ ZrchI (Figure 9B) mice could be explained by a normal α_2_M activity not compromised by the ineffective binding to PrP. When Dpl is ectopically expressed in the CNS and PrP is simultaneously *knocked-out* (Figure 9C), Dpl binding to α_2_M triggers the cerebellar granule cells and Purkinje cells degeneration, finally leading to the pathological ataxic phenotype. When *Prnp* ^0/0^ tg mice ectopically expressing Dpl in their CNS are backcrossed with full length PrP-expressing mice, PrP co-expression rescues the ataxic phenotype (Figure 9D), and this rescue is protein concentration dependent [8]. According to our results (Figure 7, Figure 8 and Tab. 1), full length PrP can bind Dpl with high affinity. Thus, when expressed at a higher level, PrP could sequester the total amount of Dpl, prevent Dpl binding to α_2_M and inhibit Dpl-induced neurodegeneration (Figure 9D, left). On the contrary, when Dpl levels exceed PrP expression, the remaining amount of Dpl unbound to PrP is still capable of binding to α_2_M, and thus can elicit neurodegeneration (Figure 9D, right).

Unlike full length PrP, N-terminally deleted PrP is incapable of rescuing Dpl-induced degeneration [34].

Additionally, we show here SPR data indicating that truncated PrP binds to Dpl with ten folds less affinity than full length PrP (Figure 8 and Table 1). According to our model, N-terminally truncated PrP binds Dpl with less efficacy than full length PrP, even if over expressed. In this case, Dpl retains its capability of binding to α_2_M and thus of triggering cerebellar degeneration (Figure 9E).

It cannot be ruled out that PrP and Dpl may also exert respectively protective and toxic functions on cerebellar neurons through distinct, non competitive, signaling pathways, and that the final cell fate might be the result of the sum of actions between separate pathways. This in turn could explain why PrP mutants like PrPΔF show similar neurodegenerative phenotype in absence of Dpl.

However, the broad biological significance of the binding between Dpl and proteinases inhibitors needs to be further investigated. At every instance, our results are the first ones beginning to sort out a presumed physiological function of Dpl. Dpl binding to proteinase inhibitors, in fact, might normally occur in tissues with normal or higher level of Dpl expression, i.e. heart, skeletal muscle, spleen and above all testes [33]. Notably, mice devoid of Dpl are sterile. Their testes are macroscopically regular, and discordant data have been reported from different groups regarding motility and number of spermatozoa [35], [36]. However, the major cause of sterility is due to an impairment of the spermatozoa in penetrating the egg's zona pellucida [35], thus suggesting an involvement of Dpl in correct spermatogenesis and sperm-egg interaction. Indeed, members of α_2_M family are found in the cytoplasm of Sertoli cells and in the tubular lumen and interstitial tissue in the testes [37], suggesting the fact the α_2_M could be a protective tool of the male reproductive system against acrosomal proteinases released during sperm maturation process. In addition, α_2_M family may play a role as a regulatory component of cytokines, growth factors, hormones and proteinases active during reproductive tissue remodeling and extracellular matrix restructuration [38].

Hence, the interaction between Dpl and α_2_M would be beneficial in testes where Dpl is physiologically highly expressed and its expression proves to be necessary for correct spermatozoa performance, while in the CNS, ectopic expression of Dpl in tg mice, and possibly its interaction with α_2_M, is highly toxic to GCL and Purkinje neurons. Thus, the same biological mechanism could be beneficial in the male reproductive tissue while lethal in the CNS.

α_2_M is a genetic risk factor for Alzheimer's disease (AD) [39], [40], [41], [42]. Moreover, α_2_M was found to co-localize with beta-amyloid (Aβ) plaques in AD patients [43], and is supposed to mediate the internalization and the clearance of α_2_M-Aβ complexes, possibly by interaction with one of its major neuronal receptors, the low-density lipoprotein receptor-related protein (LRP) [44], [45], [46], [47], [48]. Indeed, PrP internalization and trafficking is also mediated by LRP [49], [50]. This finding raises the question whether a possible interaction of PrP^C^ with LRP could have a key role in the conversion of PrP^C^ into PrP^Sc^. Interestingly, α_2_M was found to facilitate, at least *in vitro*, PrP^C^–PrP^Sc^ conversion [51]. In light of our findings, albeit α_2_M was not found to directly interact with PrP^C^, it cannot be ruled out that α_2_M activity could play a role also in prion diseases and other neurodegenerative disorders in addition to AD.

## Supporting Information

Figure S1 MASCOT Search Result output. (A) List of the 29 peptides after MALDI-TOF MS analysis matching the query. (B) Primary aminoacid sequence of rat α1I3. Matched peptides are shown in red bold.
(4.13 MB TIF)

Text S1 Recombinant mouse PrP and Dpl production and purification
(0.03 MB DOC)

Text S2 Sequence similarity of proteins belonging to α_2_M superfamily
(0.19 MB DOC)
