# Correction: Prion Protein Paralog Doppel Protein Interacts with Alpha-2-Macroglobulin: A Plausible Mechanism for Doppel-Mediated Neurodegeneration

**DOI:** 10.1371/annotation/6f581801-c5f0-4fb0-9e55-6ee35653cd12

**Published:** 2009-08-18

**Authors:** Stefano Benvegnù, Diego Franciotta, Josh Sussman, Angela Bachi, Elisabetta Zardini, Paola Torreri, Cedric Govaerts, Salvatore Pizzo, Giuseppe Legname

In addition to their currently listed affiliation, Stefano Benvegnù and Giuseppe Legname are also affiliated with: Italian Institute of Technology - SISSA Unit, Trieste, Italy. 

