# Supplementary figures and images for: Prion Protein Paralog Doppel Protein Interacts with Alpha-2-Macroglobulin: A Plausible Mechanism for Doppel-Mediated Neurodegeneration
Source: PLoS One. 2009 Jun 18;4(6):e5968. doi: 10.1371/journal.pone.0005968 (PMC2693666; doi:10.1371/journal.pone.0005968)

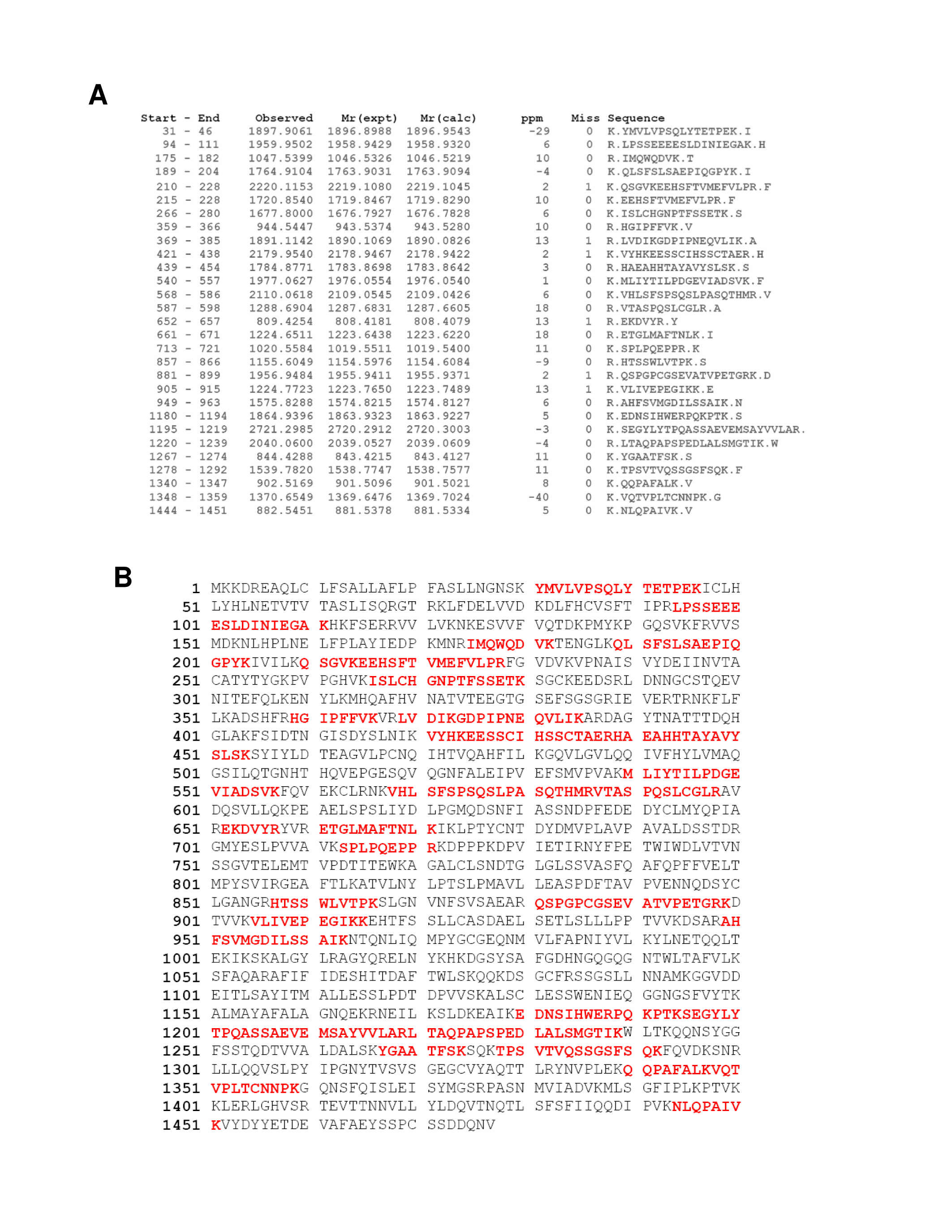

Supplement: Figure S1 — MASCOT Search Result output. (A) List of the 29 peptides after MALDI-TOF MS analysis matching the query. (B) Primary aminoacid sequence of rat α1I3. Matched peptides are shown in red bold. (4.13 MB TIF) [file pone.0005968.s001.tif]
