# Supplementary material for: Prion Protein Paralog Doppel Protein Interacts with Alpha-2-Macroglobulin: A Plausible Mechanism for Doppel-Mediated Neurodegeneration
Source: PLoS One. 2009 Jun 18;4(6):e5968. doi: 10.1371/journal.pone.0005968 (PMC2693666; doi:10.1371/journal.pone.0005968)
Supplement: Text S1 — Recombinant mouse PrP and Dpl production and purification (0.03 MB DOC) [file pone.0005968.s002.doc]

### Supporting Information

### Recombinant mouse PrP production and purification

Recombinant (rec) full-length mouse (Mo) PrP spanning from aminoacid 23 to 230, MoPrP(23-230), was expressed from the pET11a plasmid (Novagen, Darmstadt, Germany) in *Escherichia coli* (*E.coli*) BL21(DE3) in minimal media containing 100 µg/ml ampicillin. The bacterial paste was resuspended in 25 mM Tris-Hcl, 5 mM EDTA (pH 8.0) and processed twice in a Microfluidizer M-110 EH (Microfluidics Corp. Newton, MA, USA). Inclusion bodies were collected by centrifugation and solubilized in five volumes of 8 M urea, 10 mM MOPS (pH 7.0) by agitation over-night at room temperature (RT). The purification was carried out using carboxy methyl sepharose followed by C4 reverse-phase media. MoPrP was obtained ≥ 95% pure and folded predominantly in α-helical conformation as measured by mass spectrometry and CD spectroscopy, respectively.

MoPrP(89-230) was expressed in *E.coli* from plasmid pNT3A as previously described [1]. The protein product was analyzed as described above.

### Recombinant Mouse Dpl production and purification

A bacterial construct for the expression of MoDpl (26-155) in the expression vector pET11d (Novagen, Darmstadt, Germany) was generated by standard procedures. Subsequent to transformation into *E.coli* BL21(DE3) cells, fermented cultures were processed in a microfluidizer as described above and recMoDpl was further purified on a Mono S HR10/10 column (Pharmacia-Biotech, Uppsala, Sweden), and peak fractions were lyophilized.

Purity and structural conformation was assessed as described above. RecMoDpl was solubilized in either distilled water or refolded in 20 mM sodium acetate buffer (pH 5.2) at a concentration of 10 mg/ml and stored at 4°C until needed.

**References**

1. Ryou C., Prusiner S. B. and Legname G. (2003) Cooperative binding of dominant-negative prion protein to kringle domains. *J. Mol. Biol.* **329,** 323-333.
