# Supplementary material for: Prion Protein Paralog Doppel Protein Interacts with Alpha-2-Macroglobulin: A Plausible Mechanism for Doppel-Mediated Neurodegeneration
Source: PLoS One. 2009 Jun 18;4(6):e5968. doi: 10.1371/journal.pone.0005968 (PMC2693666; doi:10.1371/journal.pone.0005968)
Supplement: Text S2 — Sequence similarity of proteins belonging to α2M superfamily (0.19 MB DOC) [file pone.0005968.s003.doc]

**Sequence similarity of proteins belonging to α2M superfamily**.

Sequence alignment between rat alpha-1-inhibitor-3 (A1I3, SwissProt entry: P14046) and alpha-2-macroglobulins (A2M) of mouse (SwissProt entry: Q61838), rat (SwissProt entry: P06238) and human (SwissProt entry: P01023). The Sequence Identity between the three alpha-2-macroglobulins and rat alpha-1-inhibitor-3 is 51%, 57% and 59%, respectively. Fully conserved residues are denoted by star (*), while semi conserved are indicated by colon (:).
